# A lncRNA-miRNA-mRNA network for human primed, naive and extended pluripotent stem cells

**DOI:** 10.1371/journal.pone.0234628

**Published:** 2020-06-16

**Authors:** Zhenglai Ma, Yanni Li, Yingying Zhang, Jiaxin Chen, Tao Tan, Yong Fan

**Affiliations:** 1 Key Laboratory for Major Obstetric Diseases of Guangdong Province, Key Laboratory of Reproduction and Genetics of Guangdong Higher Education Institutes, The Third Affiliated Hospital of Guangzhou Medical University, Guangzhou, China; 2 Yunnan Key Laboratory of Primate Biomedical Research, Institute of Primate Translational Medicine, Kunming University of Science and Technology, Kunming, China; Peking University Third Hospital, CHINA

## Abstract

Human pluripotent stem cells (hPSCs) represent a promising platform for studying embryonic development, and different states of pluripotency reflect the different stages of embryo development. Here, we successfully converted three in-house-derived primed hPSC lines (H10, H24, and iPS) to a naive state and an expanded pluripotent stem cell (EPS) state. Primed, naive and EPS cells displayed state-specific morphologies and expressed pluripotent markers. The expression of SSEA4 and TRA-1-60 was downregulated in the conversion process. The H3K27me3 expression level also decreased, indicating that global methylation was reduced and that the X chromosome started to reactivate. RNA-sequencing analysis results revealed that differentially expressed genes (DEGs) were significantly enriched in both naive hPSCs and EPS cells when compared to the primed state. However, imprinted gene expression barely changed before and after state reversion. Gene ontology (GO) analyses showed that the upregulated DEGs were mostly enriched in RNA processing, DNA replication and repair, and regulation of cell cycle process, while downregulated DEGs were related to extracellular adhesion and various tissue developmental processes. Kyoto Encyclopedia of Genes and Genomes (KEGG) pathway analysis showed that EPS cells were enriched in the PI3K-Akt and Wnt signaling pathways. Analysis of the lncRNA-miRNA-mRNA competing endogenous RNA (ceRNA) network between primed, naive hPSCs and EPS cells revealed that hsa-miR-424-5p, has-miR-16-5p, has-miR-27b-3p, has-miR-29c-3p, and KCNQ1OT1 were crucial nodes with high degrees of connectivity. Our work may represent new insight into the intrinsic molecular features of different hPSC states.

## Introduction

Conventional human embryonic stem cells (hESCs) and human induced pluripotent stem cells (hiPSCs) are pluripotent cell types with the capacity to proliferate and differentiate, which makes them a critical platform for studying mechanisms for human embryo development, drug development, genome screening, cell therapies, etc. Although hESCs are derived from preimplantation human blastocysts, they typically exhibit ‘‘primed” pluripotency, in which they are morphologically and transcriptionally similar to stem cells derived from the mouse postimplantation epiblast (mEpiSCs) [1,2]. However, mouse ESCs derived from the preimplantation blastocyst can differentiate into all embryonic cell lineages *in vitro* and in a chimera model, exhibiting a ‘‘naive” state corresponding to an stage of development that is earlier than the postimplantation epiblast [1,3]. This has led to studies aimed at converting cultured human pluripotent cells into a naive state by modifying growth conditions that support self-renewal of hESCs and hiPSCs to make them akin to human preimplantation embryos. Previous studies have yielded multiple, distinct conditions and transgene-free interconversion to induce and maintain naive pluripotency [4–11]. Recently, studies have reported the establishment of human extended pluripotent stem (hEPS) cell lines featuring the molecular characteristics of blastomeres and possessing developmental potency for all embryonic and extraembryonic cell lineages [12–14]. At the same time, ‘omics’ technologies have provided unprecedented insights into the molecular complexity and heterogeneity of the human naive and primed pluripotent state [9,15–22], but to date, the extent to which the resulting cells recapitulate the *in vivo* situation and a comprehensive investigation of the expression changes in mRNAs, microRNAs (miRNAs) and long noncoding (lnc)RNAs as competing endogenous RNA (ceRNA) networks of pluripotency still await full elucidation.

Here, we adopt commercially available defined medium (RSeT^™^) and a chemical cocktail medium (N2B27-LCDM) to revert primed hPSCs to a naive state and EPS cells with extended developmental potency. RNA-Seq and bioinformatics approaches were used to comprehensively investigate the differentially expressed genes and screen crucial ceRNA interaction axes to identify different states of pluripotent stem cells. The results of this study may improve the current understanding of the molecular mechanisms involved in the transition of primed towards naive states and provide insight for stem cell studies.

## Materials and methods

### Cell culture

Human embryonic stem cell lines H10, H24 and human induced pluripotent stem cell line iPS-46 were independently established in our laboratory [23,24]. MTeSR^™^1 medium (STEMCELL Technologies), containing recombinant human basic fibroblast growth factor (rh-bFGF) and recombinant human transforming growth factor β (rh TGFβ), is a complete, serum-free, defined formulation designed for the feeder-free maintenance and expansion of hESCs and hiPSCs [25,26]. Conventional primed medium was mTeSR^™^1 [26,27], and cells were passaged every five to seven days via the dissociation of small clumps with dispase (Roche). RSeT^™^ (Stem Cell Technologies) is a defined medium for culturing naive-like human pluripotent stem cells, and cell lines use RSeT for short. EPS cells were cultured in serum-free N2B27-LCDM medium [14]. The RSeT and EPS cells were passaged every three days following the generation of a single cell suspension by treatment with TrypLE (Thermo Fisher) or Accutase (Innovative Cell Technologies).

The ratio of mTeSR^™^1 and RSeT^™^ or N2B27-LCDM was 1:1 at day 1, and then RSeT^™^ or N2B27-LCDM was used and exchanged every day while maintaining cells at 20% O_2_ and 5% CO_2_ at 37°C. By day 3 or 4, the colonies were generally large enough to be passaged. During conversion and reprogramming, the colonies began to adopt a tightly packed, highly domed morphology that is characteristic of mESCs (2i); smooth colonies gradually developed with refractive edges. The converted RSeT and EPS cells were passaged every three days following the generation of a single cell suspension by treatment with TrypLE and analyzed at approximately passage 10 after reprogramming.

### AP staining

AP activity in hPSCs was detected by a BCIP/NBT kit (BOSTER) according to the manufacturer’s instructions. Briefly, the cells were washed twice with PBS, fixed with 4% PFA/PBS (pH 7.4) for 10 min at room temperature, and washed three times with PBS. Then, the cells were incubated with a mixture (1 mL H_2_O/one drop of A/one drop of B) for 30 min at room temperature. The AP-positive colonies showed a dark violet color and were photographed with a Nikon inverted microscope.

### Karyotype analysis

The hPSCs were treated with 1.0 μg/mL demecolcine for 1.0 h at the exponential phase. The cells were then trypsinized and harvested for chromosome analysis. The chromosome analysis procedure was routinely performed as previously described [28,29].

### Flow cytometry

To determine the proportion of TRA-1-60^+^ cells, the cells were dissociated with accutase for 2 min at 37°C, filtered through a 40-μm mesh, washed two times with fluorescence-activated cell sorting (FACS) buffer (calcium- and magnesium-free PBS+5% FBS), and stained with TRA-1-60 (4746, Cell Signaling) (1:400) for 30 min on ice in the dark. The cells were washed twice with PBS and were finally suspended in 0.5 mL of FACS cell sorting buffer (calcium- and magnesium-free PBS+5% FBS+5.0 μM ROCKi), at which point TRA-1-60^+^ cells were sorted with a BD FACSAria^™^ III Cell Sorter. Cells with no fluorescence staining were used as a negative control.

### Immunostaining

To perform immunocytochemical analysis, the cells were washed twice with PBS and fixed with 4% PFA (pH 7.4) for 30 min at room temperature. Fixed cells were washed three times with PBS and were subsequently incubated with PBS containing 0.3% Triton X-100 for 1 h; then, they were blocked with FBS-BSA-blotting buffer (10% FBS, 3% BSA, and 0.3% Triton X-100) for 1 h. Then, immunostaining was performed according to standard protocols using the following primary antibodies: Oct-3/4 (R&D); SOX2 (R&D); SSEA4 (Abcam); and TRA-1-60 (Cell Signaling). Appropriate Alexa Fluor dye-conjugated secondary antibodies (Invitrogen) were used. Nuclei were stained with DAPI (Life Technologies). Images were taken using a confocal microscope (Nikon).

### Teratoma assay of Primed, RSeT and EPS cells

Primed, naive hPSCs and EPS cells were dissociated and resuspended in PBS supplemented with 30% Matrigel (Corning) and 5.0 μM Y27632. The Matrigel/PBS/cell mixture was kept on ice, as it would solidify rapidly at room temperature. We typically injected approximately 10^5^~10^6^/50 μL of hPSCs cells into mice to maximize teratoma formation efficiency [30]. The cell mixtures were subcutaneously injected into both inguinal flanks of NOD-SCID IL-2 receptor gamma null (NSG) mice. Visible teratomas formed starting at 2 weeks after injection and were collected by 8 weeks for fixation, sectioning and H.E. staining.

### Construction of libraries

Total RNA was extracted with TRIZOL^®^ (Invitrogen), and it was used for library construction employing an NEBNext^®^ Ultra^™^ RNA Library Prep Kit (NEB) according to the manufacturer’s protocol. RNA purity was assessed using a KaiaoK5500^®^ Spectrophotometer (Kaiao, China). RNA integrity and concentration were assessed using an RNA Nano 6000 Assay Kit and a Bioanalyzer 2100 system (Agilent Technologies). All cultured samples had an RIN value of > 7. A total amount of 2 μg RNA per sample was used as input material for the RNA sample preparations. Clustering of the index-coded samples was performed on a cBot cluster generation system using the HiSeq PE Cluster Kit v4-cBot-HS (Illumina) according to the manufacturer’s instructions. After cluster generation, the libraries were sequenced on an Illumina platform, and 150 bp paired-end reads were generated.

### Data analysis

The accession number for the sequencing data reported in this paper is NCBI BioProject ID: PRJNA634265. Raw reads were aligned to the reference genome (UCSC hg19) using TopHat [31] for Refseq gene annotation. Expression levels (FPKM) of Refseq genes were calculated using Cufflinks [31]. Differentially expressed genes were identified using DESeq2 [32]. Subsequently, the differentially expressed genes (DEGs) were subjected to GO and KEGG pathway analysis by using the clusterProfiler R package [33]. miRNA reads were aligned using the miRBase22 database [34]. DEG-related target genes were predicted using the starbase2.0 database [35]. Based on the miRNA-target gene interactions, lncRNAs that play regulatory roles in DEG genes were filtered and integrated, and a lncRNA-miRNA-mRNA (ceRNA) network was constructed using GDCRNATools [36] and visualized with Cytoscape [37].

### Statistical analysis

All experiments were performed with three biological replicates. Statistical analyses were performed with one-way ANOVA, which was used to study the differences between grouped data. Statistical significance was accepted at *P* < 0.05.

## Results and discussion

### Establishment of naive hPSCs and hEPS cells under RSeT^™^ and N2B27-LCDM culture conditions

**Fig 1:**
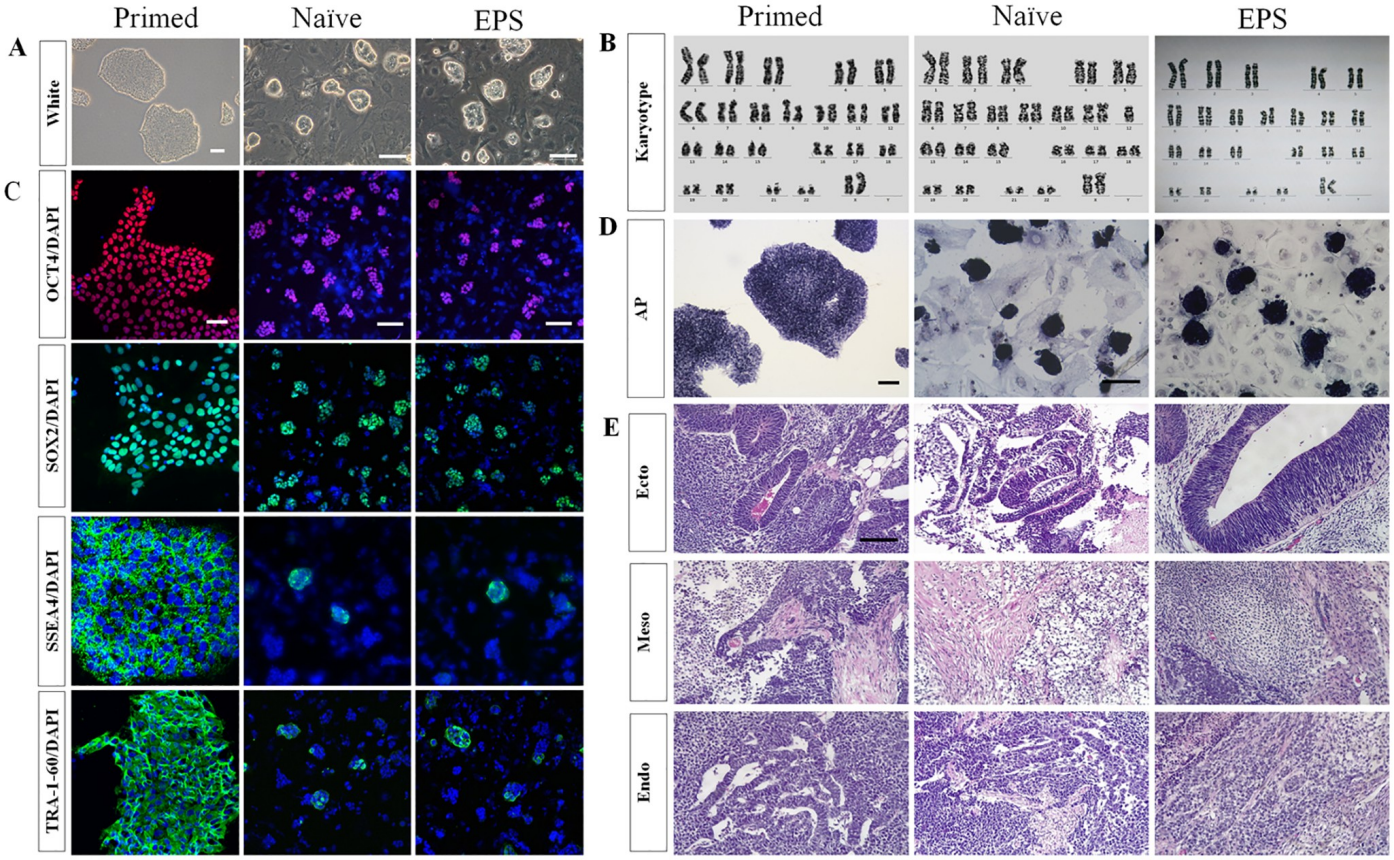
Conversion of primed hPSCs in different culture conditions. (A) Morphological analysis of hPSCs cultured in RSeT and N2B27-LCDM media and their transition from primed pluripotency-specific flat morphology towards dome-shaped undifferentiated naive colonies; (B) Chromosome analysis by colchicine shows normal karyotype of all cell lines; (C) Immunostaining for OCT4, SOX2, SSEA4 and TRA-1-60 is shown counterstained with DAPI; (D) Positive staining for alkaline phosphatase (AP); (E) Teratomas formation analysis revealed that the teratomas tissues were composed of the three germ layers; scale bar = 100 μm.

RSeT^™^ medium is a commercially available defined cell culture medium used for the reversion of primed hPSCs to a naive-like state and for the maintenance of naive-like hPSCs without bFGF or TGFβ under feeder-dependent and hypoxic conditions. N2B27-LCDM medium was developed through chemical screening, and it exhibits a broad propensity for extraembryonic and embryonic lineage differentiation [14]. Under RSeT^™^ and N2B27-LCDM cultural conditions, we successfully reprogrammed our in-house-derived primed hESCs (H10 and H24) and hiPSCs towards naive and EPS states. Before studying the expression pattern of hPSCs cells under different cultural conditions, we first characterized the different phenotypes. In contrast to primed cells that have a specific flattened morphology, the converted cells possessed high nuclear:cytoplasmic ratios and formed doom-shaped compact colonies with smooth edges; further, they could be passaged as single cells while maintaining a normal karyotype (Fig 1A and 1B), and no differences were observed among cells of different origins during the resetting process. The primed, naive hPSCs and hEPS cells exhibited positive staining for alkaline phosphatase (AP) and the specific pluripotency markers Oct4, Sox2, SSEA4 and TRA-1-60 (Fig 1C and 1D). In immunodeficient mice, three different types of hPSCs formed mature teratomas composed of the three germ layers (Fig 1E), and teratoma formation indicated that the potencies were more robust when cells were transition backward to a naive or EPS state. Therefore, we obtained naive hPSCs and hEPS cells with features resembling mouse ESCs cells by adapting a new culture medium.

### Naive hPSCs and hEPS cells showed different molecular features

**Fig 2:**
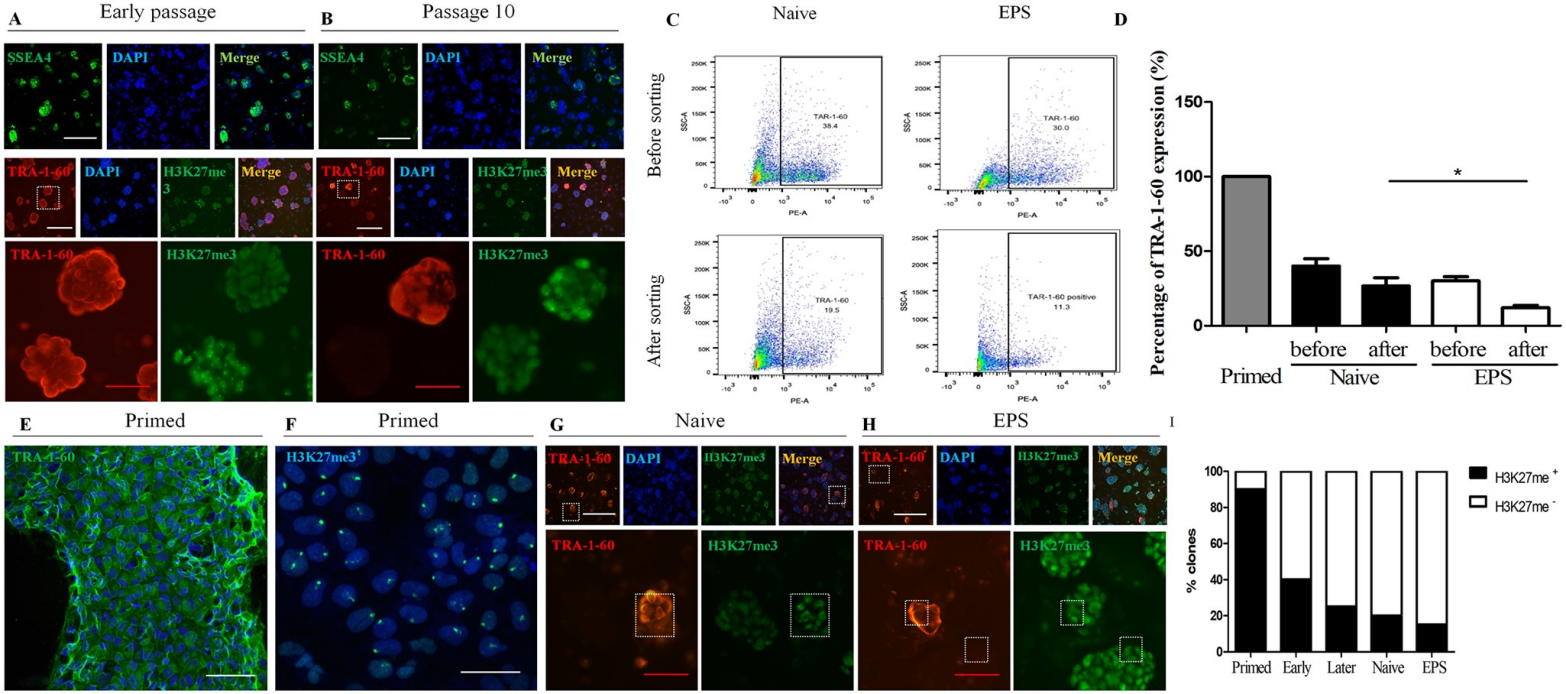
X-chromosome activation state indicated by SSEA4, TRA-1-60 and H3K27me3 expression. (A) Immunostaining of early passage cells for SSEA4, TRA-1-60, and H3K27me3. (B) Immunostaining of late passage cells for SSEA4, TRA-1-60, and H3K27me3. Reverted naive hPSCs display trends of reduced SSEA4 and TRA-1-60 expression with prolonged culture time. (C) The TRA-1-60 ratio of naive hPSCs and EPS cells before and after TRA-1-60^+^ sorting by FACS. (D) The bar chart shows the TRA-1-60 expression percentages of primed, naive and EPS cells, and the ratio was different between naive and EPS cells after sorting (*P*<0.05). (E) TRA-1-60 expression in primed cells. (F) H3K27me3 expression in primed cells. (G) TRA-1-60 and H3K27me3 expression in naive hPSCs. (H) TRA-1-60 and H3K27me3 expression in EPS cells. (I) The bar chart shows the H3K27me3-positive clone percentages of primed, naive and EPS cells. Magnified images show the staining results of dotted line marks area of low power images. Scale bars = 100 μm (F: Scale bars = 50 μm).

The epigenetic differences among primed, naive hPSCs and hEPS cells offered a good platform to study cell state changes. We discovered that reverting primed hPSCs to a naive or EPS state resulted in a decreasing and heterogeneous expression pattern for the human pluripotency surface markers SSEA4 and TRA-1-60 at later passages, which indicates that the hypomethylated transcriptional program resembled that of the human preimplantation epiblast (Figs 1C and 2A). Next, we sorted the RSeT^™^ and N2B27-LCDM cultured cells into TRA-1-60 positive and negative populations using fluorescence activated cell sorting (FACS), and then we replated the sorted cells in matched media. We found that TRA-1-60-negative cells yielded mostly domed colonies. One passage after sorting, the TRA-1-60 negative cells maintained low TRA-1-60 expression, indicating that this is a relatively stable state (Fig 2B). Furthermore, compared to primed and naive states, hEPS cells showed diminished expression of TRA-1-60 (Fig 2C and 2D, *P*<0.05).

H3K27 trimethylation (H3K27me3) can mediate DNA methylation-independent genomic imprinting, which correlates with general demethylation at the DNA level. Dynamics of H3K27me3 expression contribute to X chromosome inactivation in differentiating female pluripotent cells [38]. Naive cells and EPS cells are normally characterized by a reduction in H3K27me3 signaling at the promoters of lineage-specific genes and the gene body regions of developmental genes, and both X chromosomes are typically active in female cells relative to their primed counterparts [39]. Our results showed that along with the decreasing expression of TRA-1-60, H3K27me3 expression was reduced in naive hPSCs and hEPS cells (Fig 2E–2H). Consistent with X chromosome activation, there were significantly fewer H3K27me3 foci in reset female cells than there were in controls (Fig 2I). These data support that naive hPSCs and hEPS cells demonstrate molecular features distinct from their primed counterparts.

### Profiling of mRNA, miRNAs and lncRNAs of naive hPSCs and hEPS cells

**Fig 3:**
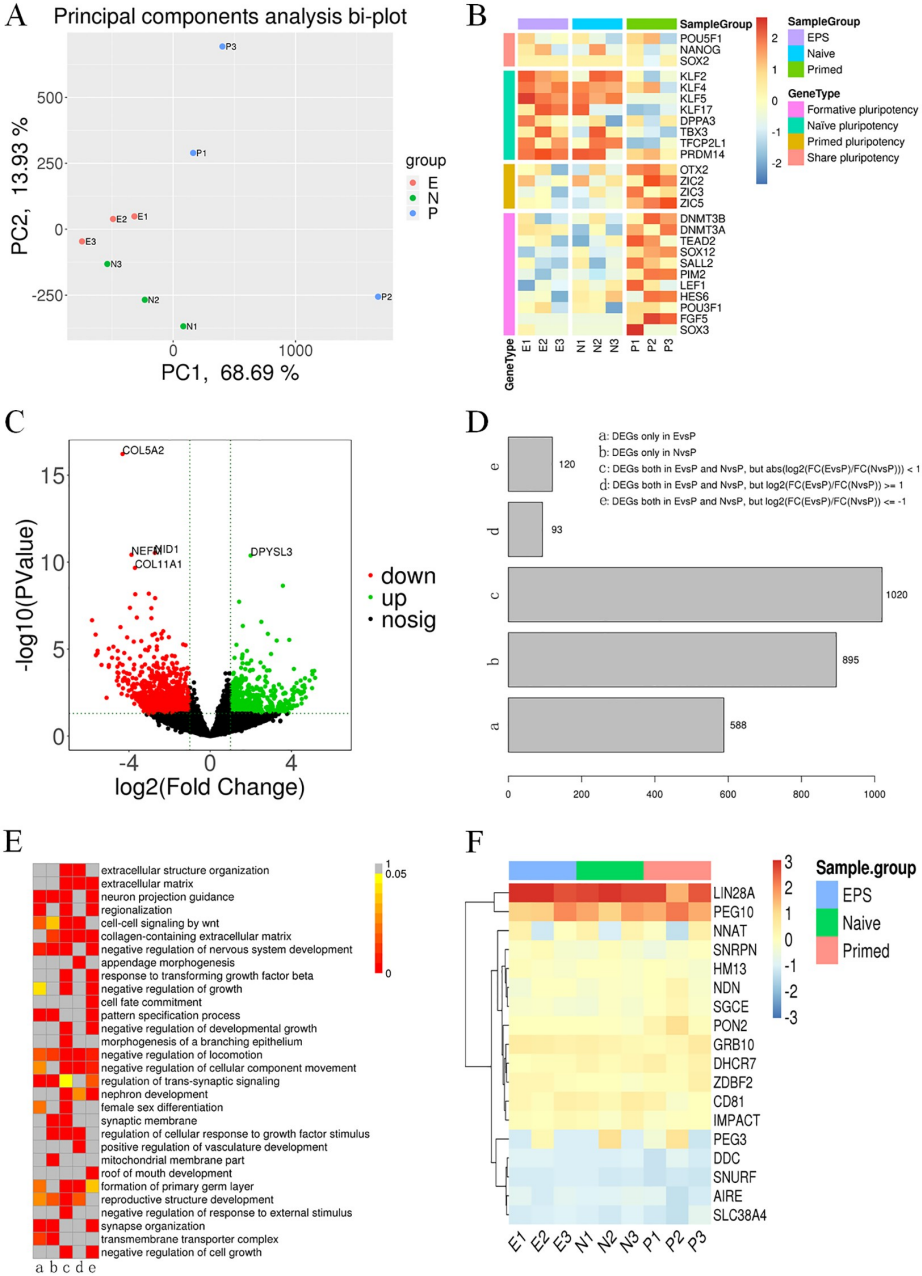
Genome-wide expression analysis of parental primed hESCs and converted naive hESCs in different conversion conditions. (A) PCA based on DEGs revealed that the transcriptomes of naive hPSCs and hEPS cells were similar. (B) Expression of genes identified by others as associated with the naive, EPS cells and primed states in humans. Expression levels, as determined by RNA-seq. (C) Volcano plot labeling DEGs. Volcano plot indicated upregulated and downregulated DEGs between EPS cells, naive cells and primed cells. Red dots represent genes with significantly downregulated expression, green dots represent genes with significantly upregulated expression, while black dots represent genes with no significant difference. (D) DEGs divided according to different groups. A and B represent the number of DEGs in naïve and EPS compared to primed hPSCs, separately. C, D, E represent comparing to primed, the number of DEGs that naïve and EPS hPSCs shared. (E) GO enrichment terms of DEGs in (D). P values are indicated in the corresponding lattice. (F) Imprinted genes reveal the change in epigenetic status between reverted cells and their origins.

We characterized the mRNA, miRNA and lncRNA transcripts by RNA sequencing from 3 naive samples and 3 hEPS cells and compared them to 3 matched primed samples at equivalent passages. Based on the threshold (*P* value <0.05 and |log2FoldChange| >1), 1963 differentially expressed genes (DEGs) were identified from mRNAs among the three different groups (S1A Fig). Principal component analysis (PCA) based on DEGs revealed that the transcriptomes of naive hPSCs and hEPS cells were similar (Fig 3A), as reflected by the close alignment of their profiles. In-depth dissection of the DEGs to identify a subset of key genes required for the acquisition of pluripotency under different conditions led to the following comprehensive observations. All groups expressed key transcription factors (POU5F1, NANOG, and SOX2) in regulating pluripotency. Naive and hEPS cells had elevated levels of KLF and reduced expression of primed state master regulators, such as the ZIC family of transcription factors and OTX2 [18]. The formative (intermediate) pluripotency stage is thought to be part of a developmental continuum between the naive and primed stages [40], and compared to primed hPSCs, naive and hEPS cells have lower expression of formative pluripotency marker genes (Fig 3B). Volcano plots showed how DEGs were distributed, and most of the DEGs were downregulated in naive and hEPS cells (Fig 3C). Then, we performed gene oncology (GO) and Kyoto Encyclopedia of Genes and Genomes (KEGG) pathway analysis on the DEGs that were divided according to different groups (Fig 3D). GO analysis showed enrichment for terms related to neuron projection guidance, collagen-containing extracellular matrix, negative regulation of nervous system development, negative regulation of locomotion and so on (Fig 3E). Pathway analysis showed ununiform enrichment in the PI3K-Akt and Wnt signaling pathways, axon guidance and other disease-related pathways (S1B Fig). We also examined the transcription levels of imprinted genes to address the epigenetic status between reverted cells and their origins. The results indicated that there were no significant changes after reversion (Fig 3F).

For miRNA expression level analysis, we identified DEGs among the three groups, and volcano plots show that they were distributed equally and that the number of DEGs from miRNAs was far smaller than that from mRNAs (S2A and S2B Fig). GO and KEGG pathway analysis showed that naive and hEPS cells have different miRNA expression patterns, and most DEGs were shared by those two groups (S2C Fig). At the same time, DEGs in lncRNA expression among naive, primed hPSCs and hEPS cells were comparable, and only a few (approximately 30 in each group) lncRNAs were differentially expressed (S2D Fig). We drew a Venn diagram based on the mRNA, miRNA and lncRNA levels of the DEGs. The results showed that EPS cells and naive hPSCs share 1233 DEGs at the mRNA level, 16 DEGs at the miRNA level and 455 DEGs at the lncRNA level, all of which means that primed hPSCs have expression patterns distinct from those of the other groups (S3A–S3C Fig). Compared to naive hPSCs, EPS cells specifically differentially expressed BEX1, DNMT3L, DPPA3, VGF, and C9orf64 at the mRNA level, AC003975.1 at the lncRNA level and 16 other DEGs at the miRNA level. These genes are mainly related to preimplantation embryos and gene expression; however, their roles in EPS cells require further validation.

### CeRNA network of naive hPSCs and hEPS cells

**Fig 4:**
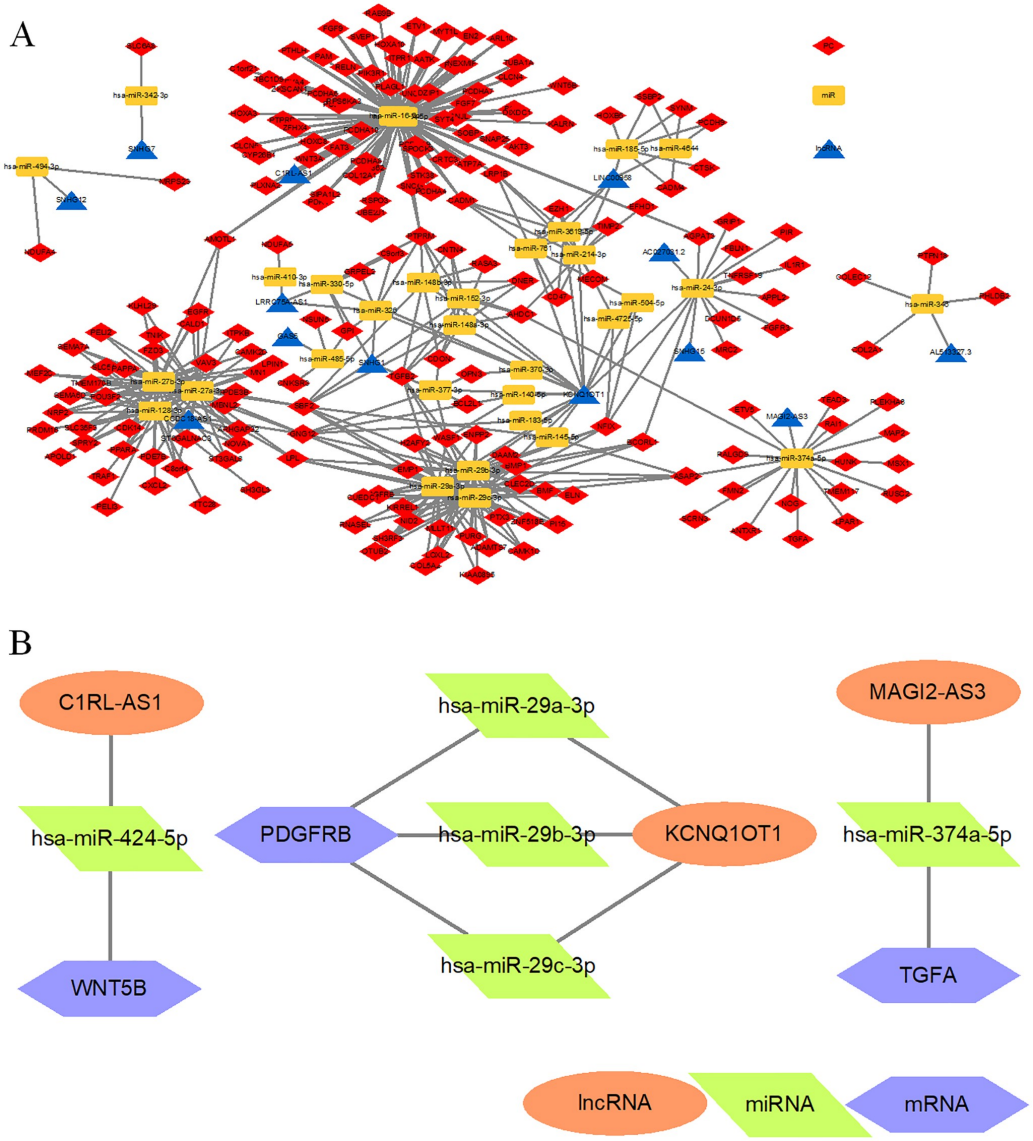
Histogram of KEGG pathway enrichment distribution of DEGs in a ceRNA regulation network. (A). CeRNA network combined with miRNA, lncRNA, and mRNA of primed, naive and EPS cells. (B). CeRNAs were subjected to the prediction of naive-primed related signaling pathways.

CeRNA regulation network analysis between primed, naive hPSCs and EPS cells was performed using GDCRNA tools [36]. Thirty-two miRNAs were predicted to interact with 195 mRNAs and 13 lncRNAs, among which hsa-miR-424-5p, hsa-miR-16-5P, hsa-miR-27a-3p, hsa-miR-27b-3p, hsa-miR-128-3p, hsa-miR-29a-3p, hsa-miR-29b-3p, hsa-miR-29c-3p, and KCNQ1OT1 were crucial nodes with high degrees of connectivity (Fig 4A). To further understand the roles of the significant RNAs described above, ceRNAs subjected to the prediction of naive-primed related signaling pathways (PI3K-Akt, MAPK and Wnt) were identified (Fig 4B). However, further investigations are required to confirm their roles in hPSCs.

Many effects have been tried to establish preimplantation-like hPSCs; however, only a few groups have met the stringent criteria exhibited by the the naive state, which has only recently been achieved by combining small molecules, growth factors and cytokines. Previous studies have identified crucial genes and pathways that drive lineage differentiation, and inhibiting these genes and pathways will allow us to obtain pluripotent cells that confer chimeric competency to both embryonic and extraembryonic tissues. In this study, we adopted commercially available RSeT^™^ medium for naïve hPSCs contains pre-screened components and does not contain bFGF or TGFβ, which were required for primed state hPSCs to maintain their pluripotency. FGF further stimulates MAPK/ERK (MEK) pathway to induce differentiation of hPSCs. In addition, wnt/ β-catenin is a key pathway during the embryo development and activate in naïve hPSCs but reduced in primed hPSCs, which can be improved by inhibiting glycogen synthase kinase-3 (GSK3). N2B27-LCDM system contain human LIF and to inhibit MEK cascade, CHIR99021 to inhibit GSK3, (S)-(+)-dimethindene maleate (DiM) and minocycline hydrochloride (MiH) to support long-term self-renewal of dome-shaped hPSCs. Further improvements in the culture methods for hPSCs and techniques for in-depth sequencing may provide comprehensive knowledge for embryo development.

Cell surface proteins with tissue-specific expression that are developmentally regulated provide a standardized and straightforward approach for defining and characterizing state-specific hPSCs [15]. SSEA4 and TRA-1-60 were used as markers to identify primed hPSCs, and we observed that their expression was downregulated and that the DNA methylation level (H3K27me3 expression) of these genes was also lower during cell state conversion. Genomic imprinting is an epigenetic process resulting in parent-of-origin specific preferential (monoallelic) expression. A previous study showed that the methylome of naive hPSCs is distinct from the human oocyte due to loss of DNA methylation at primary imprints [18]. Our results showed that the expression of most imprint genes is not changed before and after conversion, which indicates that the culture conditions may not change the expression pattern of imprinted genes. Primed hPSCs exhibited expression profiles of post-implantation human embryos. There were 2312 and 1963 DEGs in naive hPSCs and EPS cells, respectively, when compared to the primed state. A large part of genes from DEGs were also found inhuman embryonic cells from oocyte to morula stage, which reflected the recapitulation of pre-implantation state of human embryos. EPS possessed expanded potency for extra-embryonic cell lineages and KEGG analysis shows that the DEGs of EPS cells are mainly involved in the PI3K-Akt, Wnt and MAPK/ERK signaling pathways. The PI3K-Akt pathway facilitates the induction of naive pluripotency. Naive hESCs secrete Wnts to promote efficient self-renewal and inhibit transition to the primed state. MAPK is a pivotal signaling pathway involved in early embryonic development, playing roles in gastrulation, transition from the naive state to the primed state, and stabilization of primed-state pluripotent stem cells [1,41–43]. However, the intrinsic factor that mechanistically modulates the activity of MAPK/ERK needs further elucidation.

In conclusion, in-house-derived primed hPSCs were induced to recapture a naive or reset state within the same laboratory by adopting commercially available RSeT^™^ and N2B27-LCDM systems. Derived naive hPSCs and EPS cells share similarity in morphology, pluripotency and function. The intrinsic molecular and transcriptional differences of primed, naive hPSCs and EPS cells were revealed through in-depth RNA sequencing. Crosstalk between mRNAs, miRNAs and lncRNAs shows the complex mechanism underlying the reprogramming process, but further *in vitro* and *in vivo* investigations are required to confirm their roles in the pluripotency of hPSCs. In addition, EPS cells present a unique transcription profile resembling preimplantation embryos that could become a cellular platform for translational research in medical regeneration and biotechnology. Our work may provide a novel perspective for understanding the intrinsic mechanism of different states of hPSCs.

## Supporting information

S1 Fig (A) Based on the threshold (*P* value <0.05 and |log2 Fold Change| >1), about 1963 genes differential expressed genes (DEGs) were identified from mRNAs among three different groups. (B)Pathway analysis showed ununiform enrichment in PI3K-Akt and Wnt signaling pathway, axon guidance and other disease related pathways.
(DOCX)

S2 Fig (A B) DEGs among three groups and volcano plots showed their distributed equally and the number of DEGs from miRNA far more less than that from mRNA. (C) GO and KEGG pathway analysis showed naive and hEPS. (D) DEGs in lncRNA expression among primed, naive hPSC and hEPS.
(DOCX)

S3 Fig Venn diagram based on DEGs at mRNA (A), miRNA (B) and lncRNA (C) level. The results shown EPS and naive hPSCs share 1233 DEGs on mRNA level, 16 DEGs on miRNA and 455 DEGs on lncRNA level.
(DOCX)
