# Supplementary material for: A lncRNA-miRNA-mRNA network for human primed, naive and extended pluripotent stem cells
Source: PLoS One. 2020 Jun 16;15(6):e0234628. doi: 10.1371/journal.pone.0234628 (PMC7297305; doi:10.1371/journal.pone.0234628)
Supplement: S1 Fig — (A) Based on the threshold (P value <0.05 and |log2 Fold Change| >1), about 1963 genes differential expressed genes (DEGs) were identified from mRNAs among three different groups. (B)Pathway analysis showed ununiform enrichment in PI3K-Akt and Wnt signaling pathway, axon guidance and other disease related pathways. (DOCX) [file pone.0234628.s001.docx]

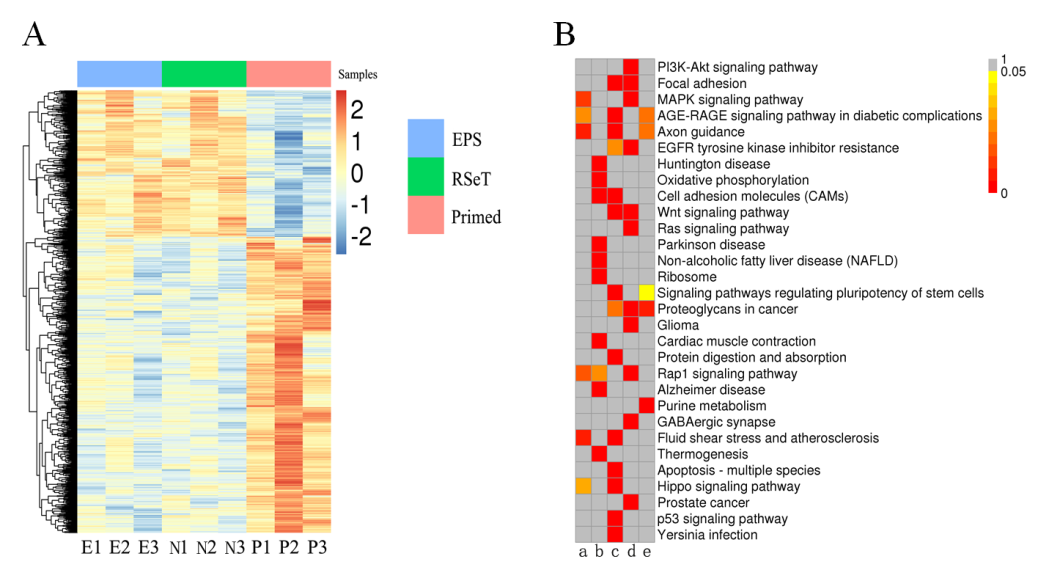


S1 Fig (A). Heatmap based on the threshold (*P* value <0.05 and |log2FoldChange| >1) about 1963 genes differential expressed genes (DEGs) were identified from mRNAs among three different groups (EPS, naive and primed). (B). Pathway analysis showed ununiform enrichment in PI3K-Akt and Wnt signaling pathway, axon guidance and other disease related pathways. (b-d). Comparsion with primed and naive groups, the EPS groups were enrichment in PI3K-Akt, Wnt signaling pathway and prostate cancer disease.

*a. DEmiRNAGs only in EvsP; b. DEmiRNAGs only in NvsP; c. DEmiRNAGs both in EvsP and NvsP ,but abs(log2(FC(EvsP)/FC(NvsP)))＜1; d. DEmiRNAGs both in EvsP and NvsP, but abslog2(FC(EvsP)/FC(NvsP))≥1; e. DEmiRNAGs both in EvsP and NvsP, but abslog2(FC(EvsP)/FC(NvsP)) ≤ 1.*
