# Supplementary material for: A lncRNA-miRNA-mRNA network for human primed, naive and extended pluripotent stem cells
Source: PLoS One. 2020 Jun 16;15(6):e0234628. doi: 10.1371/journal.pone.0234628 (PMC7297305; doi:10.1371/journal.pone.0234628)
Supplement: S2 Fig — (A B) DEGs among three groups and volcano plots showed their distributed equally and the number of DEGs from miRNA far more less than that from mRNA. (C) GO and KEGG pathway analysis showed naive and hEPS. (D) DEGs in lncRNA expression among primed, naive hPSC and hEPS. (DOCX) [file pone.0234628.s002.docx]

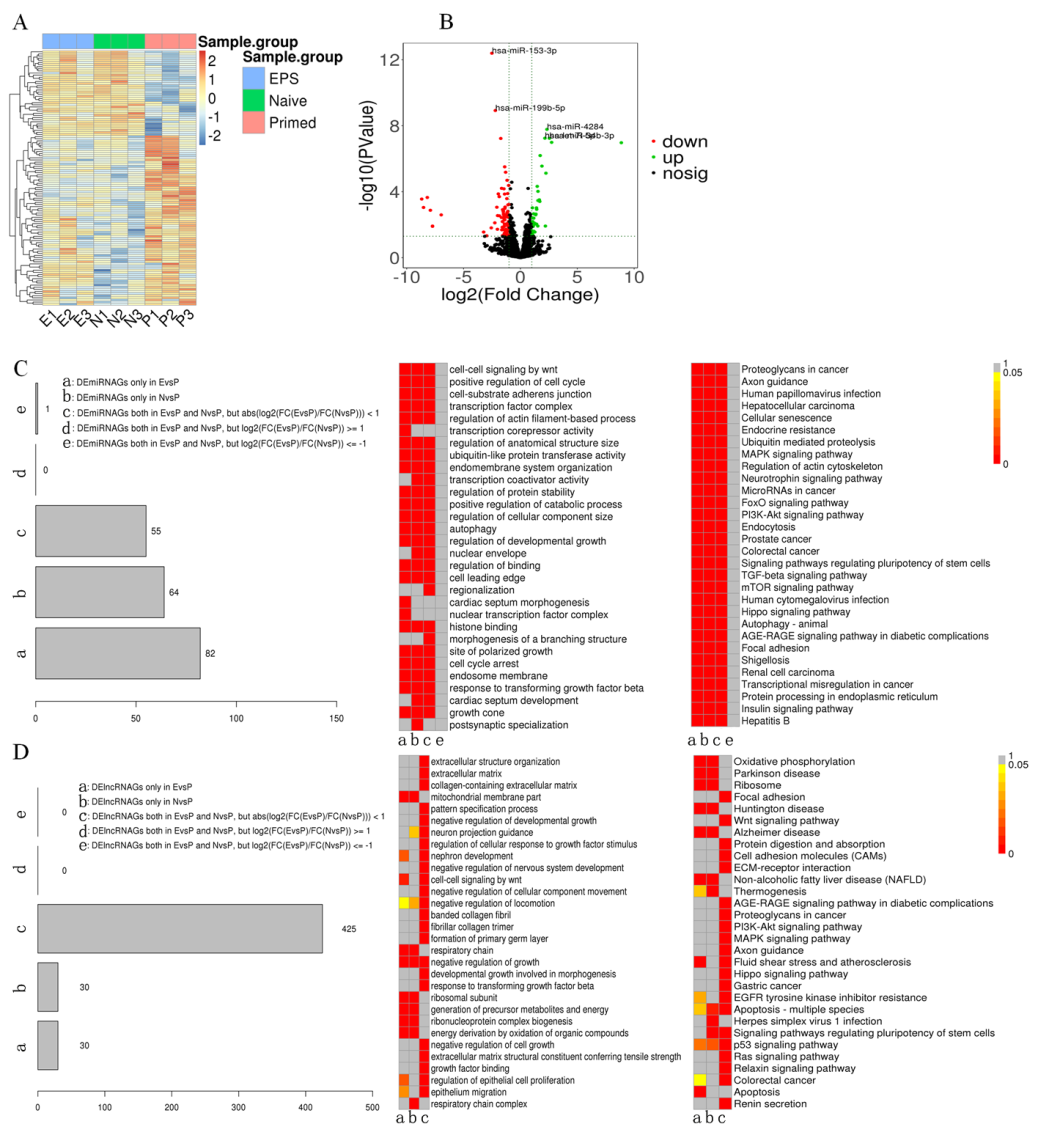


S2 Fig (A B). DEGs among three groups and volcano plots showed their distributed equally and the number of DEGs from miRNA far more less than that from mRNA. (C). GO and KEGG pathway analysis showed naive and EPS. (D). DEGs in lncRNA expression among naive, primed hPSC and hEPS.
