# Supplementary material for: A lncRNA-miRNA-mRNA network for human primed, naive and extended pluripotent stem cells
Source: PLoS One. 2020 Jun 16;15(6):e0234628. doi: 10.1371/journal.pone.0234628 (PMC7297305; doi:10.1371/journal.pone.0234628)
Supplement: S3 Fig — The results shown EPS and naive hPSCs share 1233 DEGs on mRNA level, 16 DEGs on miRNA and 455 DEGs on lncRNA level. (DOCX) [file pone.0234628.s003.docx]

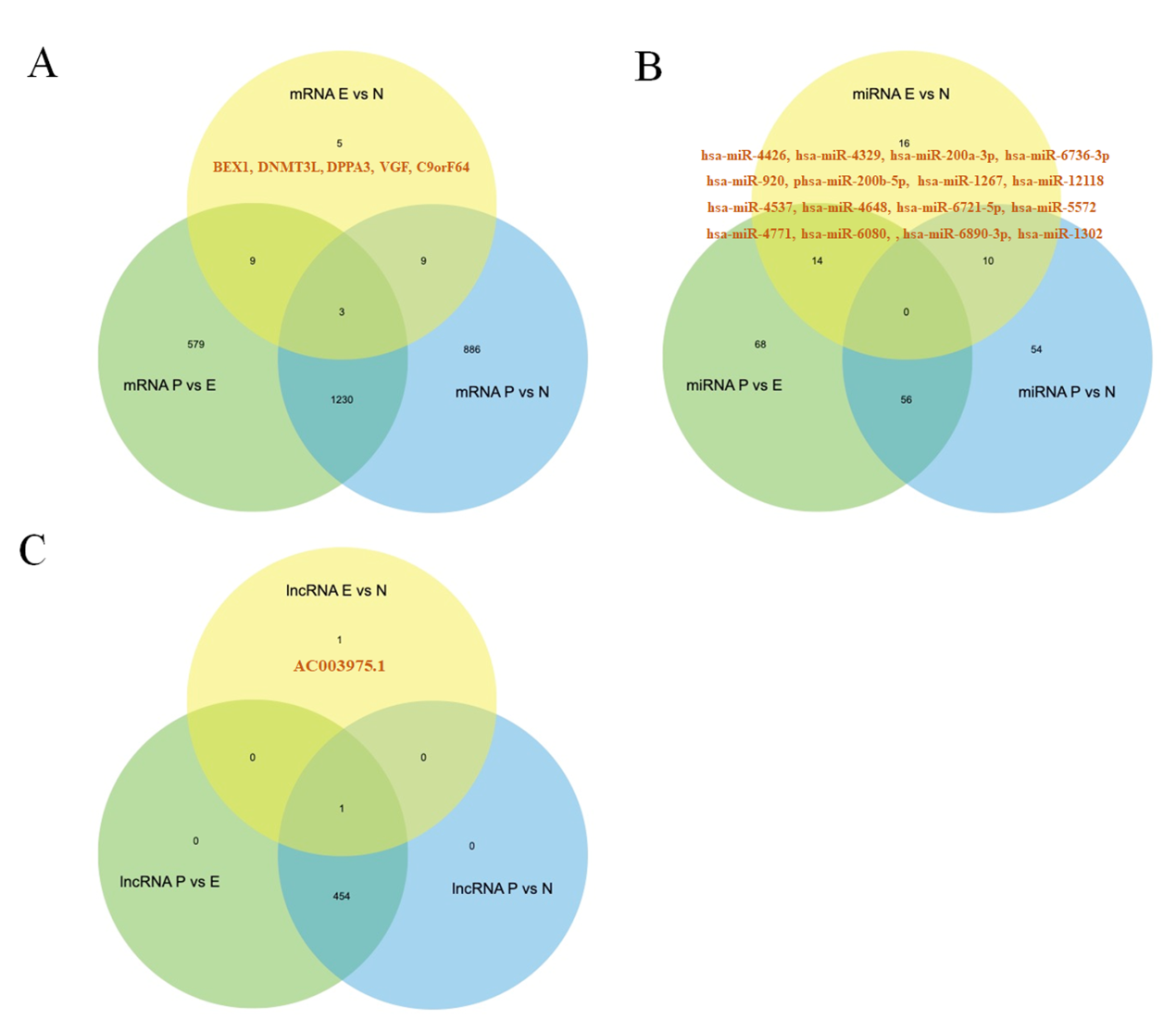


S3 Fig Venn diagram based on DEGs at mRNA (A), miRNA(B) and lncRNA(C) level. The results shown EPS and naive hPSCs share 1233 DEGs on mRNA level, 16 DEGs on miRNA and 455 DEGs on lncRNA level.
